# Perinatal depression management: A narrative review of guidelines and recommendations in chosen European countries

**DOI:** 10.18332/ejm/225591

**Published:** 2026-09-11

**Authors:** Kornelia Bartoszewicz, Ewa Sowa, Edyta Szurowska, Marlena Robakowska

**Affiliations:** 1 Student Scientific Circle Interdisciplinary Health Care Management, Department of Public Health and Social Medicine, Division of Radiology, Faculty of Health Sciences, Institute of Maritime and Tropical Medicine, Medical University of Gdańsk, Gdańsk, Poland; 2 Department of Radiology Medical University of Gdańsk, Gdańsk, Poland; 3 Department of Public Health and Social Medicine, Faculty of Health Sciences, Institute of Maritime and Tropical Medicine, Medical University of Gdańsk, Gdańsk, Poland

**Keywords:** perinatal depression, maternal mental health, healthcare policy, perinatal care, screening

## Abstract

Perinatal depression (PND) is a mood disorder occurring during pregnancy or within a year after childbirth, affecting up to 12% of women. Given its prevalence and burden, effective national strategies for screening and treatment are essential for its mitigation. This is a narrative review that examines current approaches to managing PND across fifteen European countries with a focus on the availability of national guidelines for screening in order to identify gaps in maternal mental health and recommend feasible solutions based on examples from countries implementing effective programs. The guidelines were identified through searches in PubMed, Scopus, EMBASE, general web search engines, and websites of national healthcare institutions by two independent reviewers conducting the search. Guidelines were included only if they were officially issued or endorsed by national authorities, applied nationwide, and provided specific procedures and tools for screening.

Among the fifteen countries examined, only four had national guidelines addressing both screening and treatment, and two had guidelines focused solely on treatment. Our evidence also indicates growing interest in PND, with several countries enhancing parental mental healthcare through pilot initiatives. International programs like the EU’s Riseup-PPD and the PATH project sought to strengthen perinatal mental healthcare by supporting healthcare professionals and researchers. Despite the lack of national guidelines in some countries, NGOs have created comprehensive materials and national versions of screening tools.

Stronger engagement from national health authorities is essential to ensure women receive adequate, timely care. Obligatory, standardized screening must be implemented alongside training for healthcare professionals.

## INTRODUCTION

Perinatal depression (PND) is a mood disorder that occurs during pregnancy or within one year after childbirth^1^. It is characterized by symptoms such as persistent sadness, loss of interest, low self-esteem, anxiety, irritability, feelings of guilt and humiliation, and, in some cases, hostility toward the infant^2^. Mothers affected by PND often show reduced sensitivity to their infants’ needs, adopt more negative or punitive parenting styles, and demonstrate emotional withdrawal during interactions. These behaviors can impair mother-infant bonding and have long-term adverse effects on the child’s emotional, social, and cognitive development^3,4^. Moreover, there is growing evidence that experiencing PND at their first or second birth leads to lower completed fertility^5^. PND is also associated with an increased risk of maternal mortality^6^. Risk factors include low socioeconomic status, prior psychiatric disorder, and poor social support^7^. Implementing early detection programs could detect women who are at higher risk and provide necessary support. Evidence shows that pregnant women participating in screening programs experience reductions in depression and anxiety, further demonstrating the need to implement such initiatives^8^.

Globally, the prevalence of PND is estimated at 11.9%, with higher rates in low- and middle-income countries (13.1%) compared with high-income countries (11.4%)^9^. Despite its significant prevalence and burden, up to half of all cases remain undiagnosed, which should require immediate action from healthcare authorities^10^. An extreme case is Lithuania, where out of 21753 births in 2022, only 41 women were diagnosed with postnatal depression, indicating a large gap between the actual prevalence of the condition and its diagnosis^11^. Furthermore, one study conducted in France showed that approximately 50% of parents reported that they had not received any information about their mental health after childbirth, and <40% knew whom to contact in case of mental distress^12^. Together, these findings highlight a significant lack of knowledge and awareness regarding PND.

This study reviews current approaches to managing perinatal depression across fifteen European countries that represent different regions, cultural contexts, and levels of health-care expenditure, with a focus on the availability of national guidelines for screening and treatment. The objective is to identify gaps in healthcare for pregnant women and mothers, as well as to recommend feasible solutions based on examples from countries implementing effective programs.

National guidelines for screening and treatment of perinatal depression were identified through searches in PubMed, Scopus, and EMBASE, websites of national healthcare institutions, as well as through general web search engines. Search terms included ‘PND’ or ‘PPD’ or ‘perinatal depression’ or ‘postpartum depression’ or ‘maternal mental health’ or ‘parental mental health’ AND ‘guidelines’ or ‘recommendations’ or ‘policy’ and the name of the country. To minimize bias and reduce the risk of overlooking published guidelines, two independent reviewers conducted the search, with any conflicts resolved by a third party. When these search engines yielded no results, we used additional deep search strategies to confirm the absence of guidelines. Final search was performed on 17 November 2025.

The countries were selected purposefully in order to capture the diversity of perinatal care systems across Europe. The selection took into account geographical factors (regional representation), cultural diversity (healthcare system model), and varying levels of health care expenditure.

For each included guideline, we extracted the year of initial publication along with the year of the most recent revision, the issuing authority, and the language of publication. We then recorded the scope and content of the recommendations, and the clinical settings in which screening and care were expected to take place. With respect to screening, we extracted the specific instruments named for identifying perinatal depression, together with any specified cutoff scores, recommended timing or frequency, and the personnel responsible for administering them. Where this information was not reported in the guideline itself, we noted its absence rather than inferring it from other sources. Data was extracted by two reviewers independently, and any disagreements were resolved by a third party.

## COMMENTARY

### Availability of national guidelines

**Figure 1:**
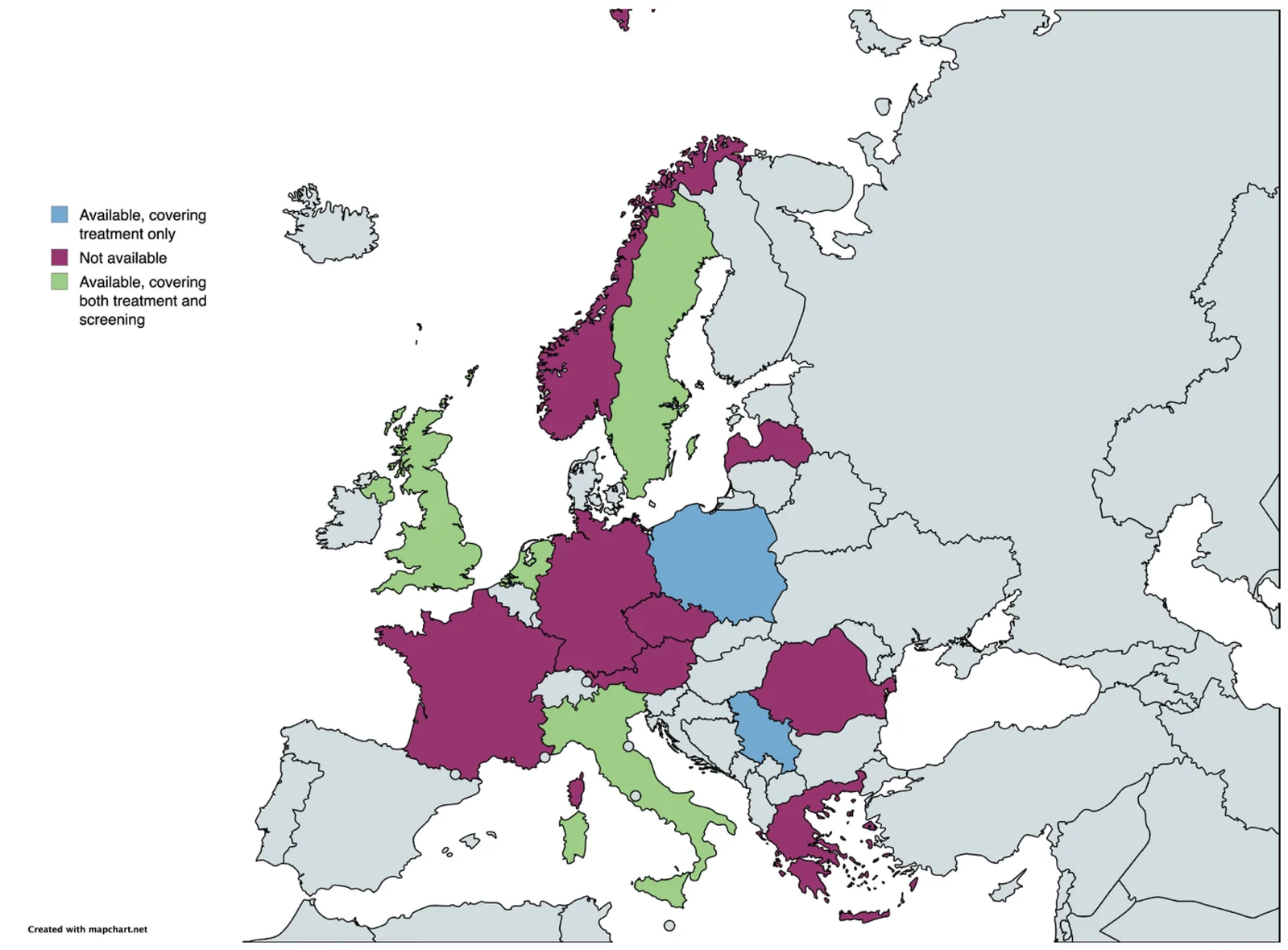
Availability of national clinical guidelines for the screening and treatment for perinatal depression across fifteen countries as of November 2025

Main findings are summarized in Table 1. Our study included fifteen representative European countries, and it highlighted that PND remains insufficiently researched and inadequately supported by national authorities^13-19^. Among these countries, eleven (Austria, Belgium, Czechia, Germany, Greece, France, Latvia, Norway, Poland, Romania, and Serbia) had no national guidelines, including screening, issued by official authorities. Serbia^17^ had guidelines published as part of a larger document covering mental disorders; however, they focused solely on treatment methods without providing practitioners with screening tools. Moreover, they were published in 2011 and thus require updating. In Poland, the 2018 Ministry of Health regulation merely requires assessing mental health in pregnant women, without specifying standardized procedures, and the Polish Psychiatric Association published guidelines regarding only the treatment of PND^16^. In Germany^13^, guidelines mention PND in dedicated chapters; however, at present, they provide no substantive information beyond indicating that the topic will be addressed. Considerable variation exists in the approaches to PND across Europe. Sweden^18^ and the UK^19^ have maintained effective programs for many years, whereas some countries lack official and substantive recommendations or have only regional programs, such as Austria and Belgium^20,21^. In Belgium^22^, comprehensive guidelines exist, yet they cover only the Flemish Region. In the Netherlands^15^, an initiative involving fifteen perinatal organizations and authorities developed and tested screening tools, e-learning for professionals, educational materials, and mapping of local support pathways. The results of this project led to national recommendations and the ‘Prevention of Postpartum Depression Toolkit’, which now form the basis of the Dutch approach to PND prevention^15^. In Norway, PND was recognized as a concern, and the need for screening was highlighted as early as 2014; however, no specific screening tools were recommended, and the use of the EPDS was not advised due to limited practitioner training^23^. Figure 1 illustrates the countries that, as of November 2025, had official, nationwide, and detailed guidelines, including recommendations for screening.

**Table 1:** Availability of national clinical guidelines for the screening, treatment, and the tools used for screening and diagnosis of perinatal depression across fifteen countries

*Country*	*National guidelines for screening*	*National guidelines for treatment*	*Is screening recommended?*	*Tool used for screening and/or diagnosis*
Austria	Not available	Not available	Not applicable	Not applicable
Belgium	Not available	Not available	Not applicable	Not applicable
Czechia	Not available	Not available	Not applicable	Not applicable
Germany	In progress [13]	In progress [13]	Not applicable	Not applicable
Greece	Not available	Not available	Not applicable	Not applicable
Italy	Available, 2025 [14]	Available, 2025 [14]	Recommended	Whooley questions, EPDS or PHQ-9
France	Not available	Not available	Not applicable	Not applicable
Latvia	Not available	Not available	Not applicable	Not applicable
Netherlands	Available [15]	Available [15]	Recommended	EPDS
Norway	Not available	Not available	Not applicable	Not applicable
Poland	Not available	Available [16]	Not applicable	Not applicable
Romania	Not available	Not available	Not applicable	Not applicable
Serbia	Not available	Available, 2011 [17]	Not applicable	Not applicable
Sweden	Available, 2010 [18]	Available [18]	Recommended	Whooley questions, EPDS
United Kingdom	Available, 2020 [19]	Available, 2020 [19]	Recommended	Whooley questions, if needed EPDS

EPDS: Edinburgh postnatal depression scale. PHQ-9: depression module of the Patient Health Questionnaire.

### International programs

The European Union also launched the Riseup-PPD program in 2019. The main goal of the initiative is to establish a Pan-European multidisciplinary network of researchers dedicated to the understanding of PND, from its prevention and assessment to its treatment and global impact^24^. Evidence-Based Clinical Practice Guidelines For Prevention, Screening and Treatment Of Peripartum Depression were published in four languages (English, German, Spanish, Portuguese) as part of the program.

The PATH (Perinatal Mental Health) project was an international initiative implemented in the Flemish part of Belgium, the Netherlands, France, and the United Kingdom, aimed at strengthening systemic mental health support during the perinatal period^12^. It sought to prevent mild and moderate perinatal mental health disorders by raising awareness, reducing stigma, and creating resources for parents and professionals. The project developed and implemented a media-based educational campaign, an international online platform with e-learning, and tools for health professionals and employers. PATH also offered face-to-face training and support groups, including programs for peer supporters, strengthening local support networks. The evaluation showed an increase in knowledge about PND, improved professional competence, and a decrease in stigmatization and depressive symptoms among participants. The project also left behind lasting resources, such as the PATH Hub platform and support networks, which can assist countries in developing their national guidelines on PND.

### Content of recommendations

The content of recommendations varies considerably between countries. Sweden^18^, the UK^19^, and Italy^14^ provide detailed, structured guidelines that direct healthcare professionals on the steps required to screen for PND and the appropriate actions to take if PND is suspected. In contrast, the Serbian guidelines mention PND only in general terms and focus more on treatment options rather than providing guidance on early detection.

Among the available guidelines, the Whooley questions are usually recommended as the initial screening step^25^. These questions are: ‘During the past month, have you often been bothered by feeling down, depressed, or hopeless?’ and ‘During the past month, have you often been bothered by little interest or pleasure in doing things?’. If a patient answers ‘yes’ to either question, further screening is recommended. This brief tool is easy to administer, does not require specialized training, and has been shown to be sensitive for detecting perinatal depression^25^. Although it may produce false-positive results in perinatal populations, it serves as a practical first step in identifying individuals who require further assessment. The Edinburgh Postnatal Depression Scale (EPDS) is commonly used as the standard tool for subsequent diagnosis of PND.

### Guidelines versus reality

Although some countries included in this study have introduced guidelines for screening, diagnosis, and treatment of PND, implementation in practice remains limited. For instance, the main issue identified in Poland is the lack of knowledge about the diagnostic procedures among family doctors and pediatricians, with waiting times for an appointment with a psychiatrist exceeding several months^26^. Moreover, the lack of psychotherapeutic care for mothers suffering from PPD and stigma around mental health hinders access to proper treatment^27,28^. In Italy, research revealed that as many as 76.3% of midwives had inadequate knowledge of PND, highlighting the need for training of medical professionals and clearer guidance^29^. Among the countries selected for this research, Sweden represents a more advanced model with clear guidelines and screening being integrated into primary care. Nonetheless, a study conducted in Sweden showed that despite universal screenings, the post-diagnostic treatment options are insufficient, as there is a limited number of specialist perinatal psychiatric services^30^. Moreover, the need for more efficient communications and screenings of immigrant mothers has been noted. In the remaining countries, such as Serbia, Romania, and the UK, there is limited empirical evidence concerning the detection and management of PND, as well as the degree of real-life implementation of guidelines. Altogether, these findings indicate that the successful implementation of national guidelines is dependent on proper training and guidance of medical professionals in terms of dealing with PND, clear referral structures, accessibility to specialists in different regions across the whole country, and ongoing monitoring.

### Role of non-governmental organizations

Despite the lack of guidelines issued by national authorities in Greece and Latvia, NGOs prepared comprehensive materials and a national version of screening tools. FAINARETI, a Greek non-profit organization, has developed its own guidelines and efforts in the area of perinatal mental health, despite the lack of official national guidelines in Greece. Since May 2014, nationwide information and education initiatives have been carried out, including a helpline, awareness campaigns, brochures, and materials for mothers and professionals, as well as the publication ‘Perinatal mental disorders: a guide for healthcare professionals’^31^. ‘Mama mums rūpi’ is a Lithuanian initiative focused on prevention and mental health support for mothers during the perinatal and postnatal periods. The organization offers, among other things, an anonymous ‘klausimynas’ (questionnaire) for the initial assessment of postnatal depression symptoms, publishes educational materials, provides contacts for free psychological help, and builds a social support network for mothers through support groups, art therapy, and regular meetings^32^.

Across fifteen European countries, this review found PND to be under-researched and inadequately supported by national authorities, with eleven lacking official nationwide guidelines that include screening. Only Sweden, the UK, Italy, and the Netherlands offered detailed, structured guidance, while others provided treatment-only, outdated, vague, or regionally limited documents. Where screening was recommended, the Whooley questions were typically the first step, followed by the EPDS for diagnosis.

### Implications

This review aims to highlight numerous critical areas for future research to improve the detection and management of perinatal depression. Electronic-based solutions represent one of the most promising solutions. Evidence suggests that eHealth interventions like online cognitive therapy can be as effective as in-person therapy, while telehealth programs can potentially alleviate the symptoms of PPD^33^. Furthermore, digital screening instruments could help overcome barriers related to stigma around mental health that prevent mothers from openly seeking help during the peripartum period. Digital tools provide us with a chance to create a safe space for struggling mothers to open up about their concerns. However, despite the rapid growth of mobile health applications, these tools still need to be of better quality and clinically evaluated to serve their purpose adequately^34^. Simultaneously, research identifying risk factors for PND should be continued and expanded. Evidence indicates that antenatal depression, low socioeconomic status, as well as high-risk pregnancy increase the risk of postpartum depression^7^. Additionally, there are promising current projects that use machine-learning and deep-learning algorithms to predict high-risk cases, and further research such as these should be considered^31,35^. Additional research should also examine the structure of different healthcare systems and factors affecting them, such as staff shortages, difficulties in referrals to specialists, and regional inequalities, to determine what effect these factors have on the effectiveness of screening for PND. Large, long-term studies involving multiple countries that combine clinical and sociodemographic data could enable a more profound understanding of risk factors and needs for treatment.

### Limitations

Only fifteen countries were included in this preliminary study, and future research should aim to expand the number of countries analyzed to provide a more comprehensive overview of PND across Europe. Moreover, comparisons between countries are challenging due to differences in their healthcare systems. It is important to note that not all relevant documents may have been identified, despite exhaustive searches. Furthermore, the prevalence figures cited in this study were derived from individual studies conducted on relatively small samples of women, often within a single hospital, clinic, or city. As such, these data may not be fully representative of the national population.

## CONCLUSION

Stronger engagement from national health authorities is essential to ensure that women receive adequate and timely care, especially given that perinatal depression is not a rare condition. National health authorities should implement standardized screening as obligatory, train healthcare professionals, and ensure equitable access to specialized care. Furthermore, the gap between the existence of national guidelines and their actual implementation must be acknowledged, as simply having such documents does not guarantee effective practice. While international efforts are needed, they should be adjusted to particular countries due to variability in healthcare systems. For instance, in Poland^36^, gynecologists are the primary care providers for pregnant women, whereas in Sweden, a midwife is the first point of contact; thus, these disparities should be addressed.

## Data Availability

The data supporting this research are available from the authors on reasonable request.
